# *Clostridium difficile* and gut health: Bacteria, the gut microbiome, and diet

**DOI:** 10.1002/imo2.50

**Published:** 2024-12-31

**Authors:** Hao Yang, Lamei Wang, Javier A. Villafuerte Gálvez, Xinru Ma, Hua Xu, Shengru Wu, Junhu Yao, Xinhua Chen, Yangchun Cao

**Affiliations:** ^1^ College of Animal Science and Technology Northwest A&F University Yangling China; ^2^ Division of Gastroenterology, Department of Medicine Beth Israel Deaconess Medical Center, Harvard Medical School Boston Massachusetts USA

**Keywords:** Clostridium difficile infection, gut microbiota, nutrients, gut metabolites

## Abstract

*Clostridium difficile* infection (CDI) is a growing global health threat, presenting significant challenges to public health. Advances in metabolomics and proteomics have enhanced our understanding of human physiology, particularly the influence of gut microbiota and dietary components on diseases like CDI. This review explores recent insights into CDI pathogenesis, including the infection cycle, toxin mechanisms, spore structure, germination, and therapeutic strategies targeting spore formation and germination. We also examine the role of gut microbiota and metabolites in CDI progression, along with the impact of diet on disease pathology through microbiota modulation and immune regulation. We aim to guide the development of precise, individualized dietary strategies to regulate gut microbiota and immune responses, offering innovative therapeutic options for CDI prevention.

## Highlights

- This review investigates the potential mechanisms of *Clostridium difficile* infection (CDI), including the life cycle of *Clostridium difficile*, and its pathogenicity in the host.
- This review explores the interactions between the gut microbiome and gut metabolites, and their role in CDI progression.
- This review explores dietary modulation as a novel approach to restore gut health and mitigate CDI severity.

## Graphical abstract

This review explores the mechanisms underlying *Clostridium difficile* infection (CDI), focusing on the pathogen's life cycle and its pathogenicity within the host. It further explores the interactions between the gut microbiome, gut metabolites, and CDI. Additionally, the review highlights dietary modulation as a promising strategy to restore gut health, highlighting its potential to alter microbiome composition and reduce infection severity.

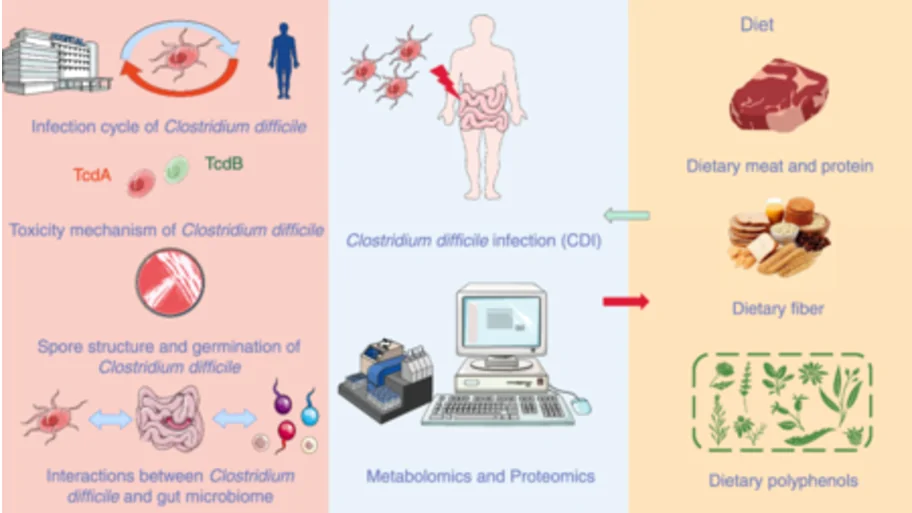

## 1 INTRODUCTION

*Clostridium difficile* infection (CDI) is caused by *Clostridium difficile* that predominantly infects the human gastrointestinal tract, particularly in patients undergoing prolonged antibiotic treatment. This infection often results in severe and potentially life‐threatening diarrhea [1]. The clinical manifestations of CDI range from self‐limiting diarrhea to more severe conditions such as pseudomembranous colitis, weight loss, malnutrition, colonic perforation, and death [2]. *Clostridium difficile* is ubiquitous, found in soil, water, and the feces of humans and animals [3]. CDI induces a range of intestinal pathologies, including colonic inflammation, water accumulation in intestinal segments [4], shortening of intestinal villous structures, increased intestinal permeability, and disruption of barrier function [5]. Over the past decade, CDI has emerged as a significant global healthcare‐associated infection. In the United States alone, over half a million CDI cases are reported annually, with approximately 29,000 associated deaths [6]. Furthermore, the economic burden of CDI is substantial. A 2018 study estimated average costs at $13,476 per case, with out‐of‐pocket expenses of $396 [7]. While antibiotic therapy effectively resolves infections in approximately 95% of CDI patients, it also disrupts the gut microbiota, leading to dysbiosis. This dysbiosis increases susceptibility to recurrent *Clostridium difficile* infection (rCDI) and other diseases in 15‐30% of individuals [8, 9, 10]. Broad‐spectrum antibiotics disrupt the gut microbiota, suppressing the indigenous microbiota and reducing colonization resistance against *Clostridium difficile*, thereby facilitating disease onset [11]. rCDI has become a significant clinical challenge, associated with a high mortality rate [12]. Addressing this issue requires a comprehensive understanding of CDI pathogenesis, improved therapeutic strategies, and a focus on mitigating gut microbiota disruption.

Fecal microbiota transplantation (FMT) is an emerging therapeutic strategy aimed at restoring gut microbiota balance by transferring processed fecal material from healthy donors to patients. It has proven particularly effective in the treatment of recurrent CDI [13]. Recent evidence highlights the critical interplay between dietary nutrient levels and the gut microbiota in shaping the gut environment. This interaction not only protects against *Clostridium difficile* colonization but also influences susceptibility to CDI and disease severity [14, 15]. Research has shown that dietary patterns profoundly impact the composition and function of the gut microbiota, leading to rapid changes within just 1 week [16]. Unhealthy dietary habits can disrupt microbiota balance, reduce resistance to bacterial colonization, and increase the risk of [17, 18]. Dietary patterns are increasingly recognized for their role in the prevention and treatment of various diseases, including cardiometabolic diseases, cancer, and inflammatory bowel disease (IBD) [19, 20]. Emerging data suggest that dietary patterns influence CDI outcomes through multifactorial mechanisms. These include alterations in gut microbiota and metabolites, regulation of the intestinal barrier and immunity, and influence on *Clostridium difficile* spore production and colonization [16, 21]. We aim to provide a comprehensive analysis of the adverse effects and underlying mechanisms contributing to CDI pathogenesis. Furthermore, it will explore how dietary interventions can be strategically utilized in therapeutic approaches for CDI management, offering a novel dimension to combat this challenging infection.

## 2 OVERVIEW OF *CLOSTRIDIUM DIFFICILE* INFECTION

### Clostridium difficile lifecycle

**FIGURE 1:**
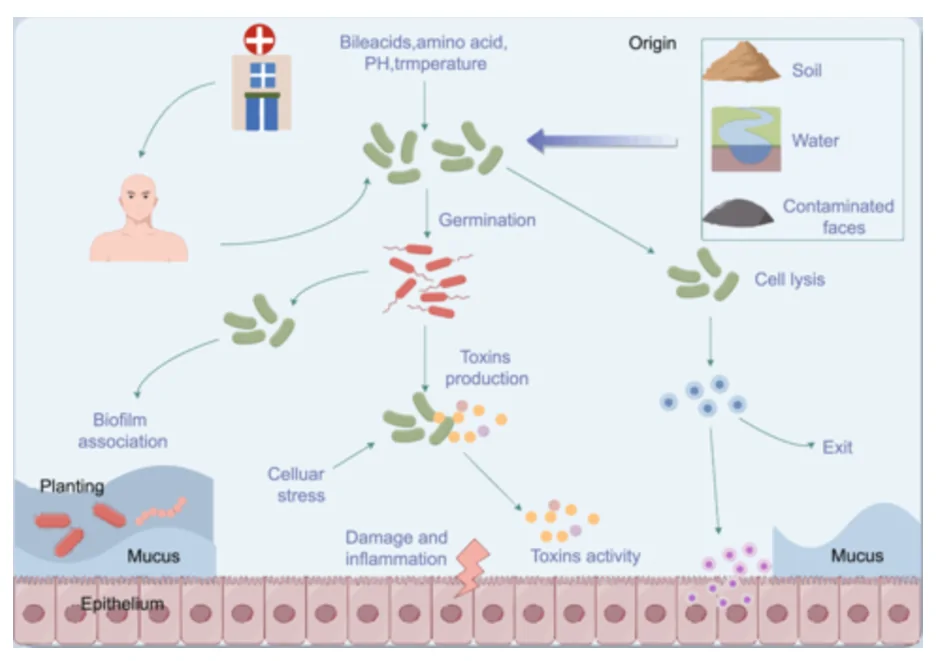
The infection cycle of Clostridium difficile. *Clostridium difficile* is primarily transmitted in clinical settings, particularly among patients undergoing antibiotic therapy for other conditions, leading to intestinal dysbiosis. Simultaneously, spores are ingested via the oral cavity, and under appropriate conditions, they germinate and produce toxins. Subsequently, as the growth phase of *Clostridium difficile* concludes, the bacterium produces spores and releases them into the intestinal environment. Some spores are excreted in the host's feces while others persist and proliferate within the intestine.

CDI is a global epidemic that predominantly affects patients with prior antibiotic exposure. Approximately 70% of CDI cases occur in healthcare settings, including hospitals and long‐term care facilities [22], while the remaining 30% of CDI cases are acquired without prior exposure to antibiotics, primarily due to the emergence of highly virulent strains in North America and Europe [23]. CDI transmission begins with the oral ingestion of spores from contaminated sources, including water, soil, or feces of other patients. These spores are protected by a multi‐layered structure that enables them to survive in the gastrointestinal tract. Once in the colon, the spores germinate, forming vegetative cells that initiate CDI [24]. Environmental signals such as bile acids, amino acids, pH, and temperature facilitate spore recognition and germination, promoting *Clostridium difficile* colonization in the intestinal tract [25, 26]. Spore germination is particularly effective in patients with dysbiosis, where the disrupted gut microbiota provides ecological niches for *Clostridium difficile*. However, commensal microorganisms can compete with *Clostridium difficile* by metabolizing nutrients and producing inhibitory byproducts [1]. Sporulation marks the transition of *Clostridium difficile* to its next lifecycle stage, which is critical for transmission and recurrence of CDI. Infected individuals excrete *Clostridium difficile* in their feces, releasing it into the aerobic environment outside the body [27]. While *Clostridium difficile* vegetative cells are strictly anaerobic and struggle to survive in aerobic conditions, spores remain viable due to their protective structure and low metabolic activity, allowing them to persist in the environment until re‐ingestion occurs [28]. Similarly, these spore characteristics pose challenges in completely eradicating *Clostridium difficile* during antibiotic therapy, contributing to the recurrence of CDI [29]. Castro‐Córdova et al. suggested that the penetration of *Clostridium difficile* spores through the intestinal barrier may contribute to spore persistence, leading to rCDI [30]. However, this theory does not fully explain why 20‐50% of rCDI cases are caused by new strains, necessitating further research [31]. Current clinical treatments aim to disrupt the CDI lifecycle at various stages. Strategies such as spore removal or inhibition of germination are crucial for CDI prevention and management. These approaches also represent promising directions for the development of novel therapeutic options in the future (Figure 1).

### Clostridium difficile spore biology

*Clostridium difficile* spores are composed of up to seven structural layers, arranged from the innermost core to the outermost exosporium. These layers include the spore core, inner membrane, germ cell wall, cortex, outer membrane, coat, and, in some strains, the exosporium. This complex structure grants the spores remarkable resistance to heat, desiccation, disinfectants, and ultraviolet light [28]. The outer membrane is encased by a protein coat, which consists of morphogenetic proteins and is assembled in an orderly fashion into a variety of proteins. These proteins self‐assemble into a robust protein coat, creating a network of interactions that confer resistance to commonly used decontaminants [32, 33]. Although not all strains of *Clostridium difficile* possess the exosporium, recent studies suggest that this structure may play a crucial role in spore‐host interactions. Specifically, the exosporium may disrupt macrophage phagosomal membranes, inducing cytotoxicity [34, 35, 36]. Spore germination in *Clostridium difficile* is governed by multiple mechanisms. Extracellular germinants are recognized by germination receptors (GRs), triggering cortex hydrolysis. Germinants bind to GRs, releasing monovalent cations and initiating the exchange of dipicolinic acid (DPA) for water. This process forms Ca‐DPA complexes, leading to water absorption, metabolic activation, and regeneration into a vegetative cell [37]. Key proteases regulate germination, notably the Csp family of (pseudo) proteases (CspA, CspB, and CspC) and the cortex hydrolase SleC [38]. Among these, the CspC pseudoprotease is critical, as its deletion results in failure to germinate [39]. CspA regulates CspC expression during spore formation, indirectly promoting germination [40]. Upon receiving external signals, CspB degrades precursor peptides that inhibit SleC, allowing SleC to activate and degrade the spore cortex, thus inducing spore germination [41]. Understanding the mechanisms underlying spore germination is essential for addressing disease transmission and treatment strategies. Further research into the precise molecular interactions between CspA and CspC could lead to novel therapeutic interventions targeting this critical stage of CDI.

#### Treatment strategies for CDI based on spore sporulation and germination

The standard treatment for CDI typically involves antibiotics such as vancomycin, metronidazole, or fidaxomicin. However, these antibiotics do not completely eliminate spores from the colon, nor do they prevent spore persistence in the environment. Additionally, conventional hospital cleaning agents are ineffective against spores, contributing to rCDI and ongoing health risks [9, 42, 43]. Therefore, it is critical to develop therapies that specifically target their removal and inhibit germination.

New antibiotics with dual activity against *Clostridium difficile* and its spores are under development. Cadazolid, a novel fluoroquinolone‐oxazolidinone antibiotic, has demonstrated significant inhibitory effects on spore germination in vitro. Clinical trials indicate that cadazolid achieves cure rates comparable to vancomycin while exerting minimal disruption to intestinal flora, including beneficial species such as *Anaplasma spp*. and *Lactobacillus spp*. [44, 45]. Another promising candidate is ramoplanin, a glycolipodepsipeptide antibiotic [46]. In vitro studies show that ramoplanin consistently and significantly reduced *Clostridium difficile* spore counts over a 28‐day period with a half‐hour treatment duration [47]. Furthermore, the removal of the spore's exosporium diminishes ramoplanin's inhibitory effect, suggesting that the drug binds to the exosporium and exerts its action during germination [47]. Therefore, ramoplanin is considered a promising drug for the treatment of CDI due to its potential to eliminate spores, which is pending further clinical trials.

In addition to antibiotics, various natural products have demonstrated the potential to inhibit the germination and formation of *Clostridium difficile* spores. Researchers have been investigating various natural compounds and extracts for their antimicrobial properties against *Clostridium difficile* [48]. For example, Roshan et al. evaluated 22 natural products and identified three (fresh onion bulb extract, coconut oil, and fresh ginger rhizome extract) that inhibited spore formation. Additionally, four natural products (artichoke extract, fresh onion bulb extract, leptospermum honey, and allicin) significantly inhibited spore growth [49]. Carvacrol, a plant‐derived extract from foods and spices, has also shown efficacy against *Clostridium difficile*. In vitro studies reported a minimum inhibitory concentration (MIC) of 1.2 mmol/L and a sub‐inhibitory concentration (SIC) of 0.6 mmol/L. At the SIC, carvacrol significantly reduced spore production and downregulated spore‐related mRNA expression, while at the MIC, it completely arrested spore growth [50]. Moreover, a combination of natural antimicrobials 20 mmol/L nisin and 0.2 mmol/L lysozyme effectively inhibited spore germination in broth medium, even when germination was stimulated by agents such as taurocholic acid and co‐germinants (trypsin and NaCl). The inhibitory effect of this combination was attributed to the disruption of the spore coat's peptidoglycan layer, allowing penetration and DNA damage within the spore core [51].

Despite current knowledge regarding strategies aimed at inhibiting *Clostridium difficile* spore germination and sporulation, their clinical application remains limited. Therefore, it is essential to enhance our understanding of the microstructure and germination mechanisms of *Clostridium difficile* spores to develop effective treatments for CDI.

#### The metabolic capacity of *Clostridium difficile* for nutrients

Clostridium difficile's survival in the gut relies on its ability to compete for resources within the host's intestinal environment and efficiently acquire essential nutrients. This metabolic flexibility enables *Clostridium difficile* to thrive, proliferate, and contribute to disease. Therefore, clarifying the relationship between nutrient availability and CDI is essential.

Simple sugars, such as maltotriose, maltose, α‐d‐glucose, d‐fructose, and d‐mannose, serve as primary carbon sources, supporting the rapid growth of all *Clostridium difficile*. Utilization of these metabolized carbon sources significantly reduces toxins production [52, 53]. However, carbohydrate‐rich diets promote gut microbial dysbiosis and increase *Clostridium difficile* colonization, which leads to higher mortality rates. This is attributed to the bacterium's rapid consumption of carbon sources, which facilitates colonization and sustained toxin production [54]. Amino acids are also essential for *Clostridium difficile*, enabling the production of ATP through substrate‐level phosphorylation via Stickland metabolism. Specific amino acids, such as proline, are preferentially utilized, enabling efficient ATP production and supporting rapid growth. This selective utilization enhances the bacterium's ability to dominate intestinal niches [55, 56]. *Clostridium difficile* also exploits host‐derived nutrients, such as ethanolamine, a type of phosphatidylinositol found in host cells. Toxin production by *Clostridium difficile* induces cellular damage and inflammation, which results in the release of ethanolamine into the gut, providing energy for the bacterium [57]. Additionally, mucins‐glycosylated proteins in the intestinal wall are degraded by symbiotic microbes like *Akkermansia muciniphila* and *Ruminococcus torques*, releasing mannose and sialic acid, which *Clostridium difficile* readily utilizes for growth [58]. Sorbitol, another host‐derived nutrient, is produced by the upregulation of aldose reductase in host epithelial cells during *Clostridium difficile*‐induced inflammation. Sorbitol promotes bacterial colonization, but its absence triggers increased toxin expression, exacerbating the inflammatory response [59]. Nutrients are crucial for the growth and colonization of *Clostridium difficile*, and their availability strongly correlates with the severity of CDI. To develop effective clinical nutritional strategies for modulating inflammation and limiting *Clostridium difficile* colonization, further investigation into its nutrient metabolism is required.

## 3 CLOSTRIDIUM DIFFICILE AND GUT HEALTH

### *Clostridium difficile* toxins activate host immune against CDI

The pathogenicity of *Clostridium difficile* in causing colitis is determined by several factors, including its virulence, including toxins encoded within the pathogenicity locus and factors associated with adherence and motility, such as adhesins, flagella, and biofilms. Not all individuals with CDI exhibit symptoms, which may be attributed to variations in *Clostridium difficile* strains. These strains are classified as toxigenic or non‐toxigenic based on their ability to produce toxins. Toxigenic strains produce toxins that, upon release in the colon, trigger inflammation, leading to colitis. This inflammation creates an environment conducive to bacterial growth and nutrient acquisition [60, 61, 62, 63].

CDI pathogenesis primarily involves two major exotoxins:: enterotoxin Toxin A (TcdA), which contributes to the destruction of the intestinal mucosa and blood vessels, and cytotoxin Toxin B (TcdB), which plays a pivotal role in disrupting the actin cytoskeleton and microtubules [64, 65]. Both toxins bind to specific receptors on host cell surfaces, including frizzled proteins (FZD), glycosaminoglycans, the low‐density lipoprotein receptor, and Toll‐like receptor 2 (TLR2). Following receptor‐mediated endocytosis, the toxins are internalized [66, 67, 68]. Within the colonic epithelium, TcdA and TcdB inactivate RhoA and Rac1, leading to cytoskeleton disruption, degradation of tight junction proteins (claudin, occludin, and ZO‐1), and severe epithelial barrier damage [69]. These toxins also inhibit Rho activity, activate the inflammasome, and recruit inflammatory cells, such as innate lymphoid cell types 1 and 3 (ILC1 and ILC3), to the colonic lamina propria, amplifying the inflammatory response [65, 70]. Some strains of *Clostridium difficile* also produce a third toxin, known as binary toxin (CDT), which may contribute to hypervirulence. However, strains that produce only CDT have rarely been detected in the human gut or feces [71]. However, the precise role of CDT in CDI pathogenesis remains to be fully elucidated [72].

The host's innate immune response caused by TcdA and TcdB is an important pathogenic mechanism of CDI. These toxins activate innate immune cells, including macrophages and neutrophils, prompting the release of pro‐inflammatory cytokines and chemokines, that recruit additional immune cells to the site of infection. This response not only combats the infection but also supports tissue homeostasis by promoting epithelial repair and mitigating inflammation‐induced damage [73]. Hasegawa et al. reported that the specific knockout of certain innate immune cytokine genes, such as interleukin 10 (IL‐10), IL‐22, and Myeloid Differentiation Primary Response Protein 88 (MyD88), resulted in worsened intestinal disease and increased mortality in CDI mouse models, highlighting the protective role of innate immunity in CDI [73, 74]. Moreover, TcdA and TcdB synergistically enhance IL‐23 expression through the MyD88 signaling pathway, underscoring the interleukin family's complex role in immune regulation [75]. As research progresses, understanding the dual roles of the interleukin family in the development of CDI (Table 1).

**TABLE 1:** Correlation between interleukins and CDI in vitro and in vivo models.

Interleukins	Model	The effects of the interleukins in CDI	Reference
IL‐22	Mice	Function of the complement system ↑; mortality ↓; numbers of pathobionts in extraintestinal organs ↓. complement‐mediated phagocytosis ↑; the level of complement C3.	[74, 76]
IL‐27	Human; mice	IL‐27 level in human and mice during CDI ↑ ; IL‐10 level ↑; IL‐17A and IL‐23 levels ↓; mortality ↓; tissue pathology ↓.	[77]
IL‐17A	Mice	Neutrophil recruitment to inflammatory sites ↑; mortality ↓; tissue pathology ↓; mucosal barrier protection ↑.	[78]
IL‐1β	Mice	Susceptibility of CDI ↓; body weight ↑; the expression of NLRP3 ↑ ; numbers of pathobionts in extraintestinal organs ↓; neutrophil recruitment to inflammatory sites ↑.	[79]
IL‐23	Mice	Mortality ↓; body weight ↑; proliferation of TH17 cells ↑.	[80]
IL‐10	Mice	The expression of IFN‐γ ↑; tissue pathology ↓; body weight ↑; clinicopathological scores ↓.	[81]

*Note*: “↑“ in the table means the quantity is increased or the effect is enhanced, “↓“ is the opposite. *Clostridioides difficile* infection (CDI); interleukin‐22 (IL‐22); interleukin‐27 (IL‐27); interleukin‐17A (IL‐17A); interleukin‐23 (IL‐23); interleukin‐1B (IL‐1B); NOD‐like receptor protein 3 (NLRP3); T helper 17 (TH17); interleukin‐10 (IL‐10); interferon‐gamma (IFN‐γ).

**FIGURE 2:**
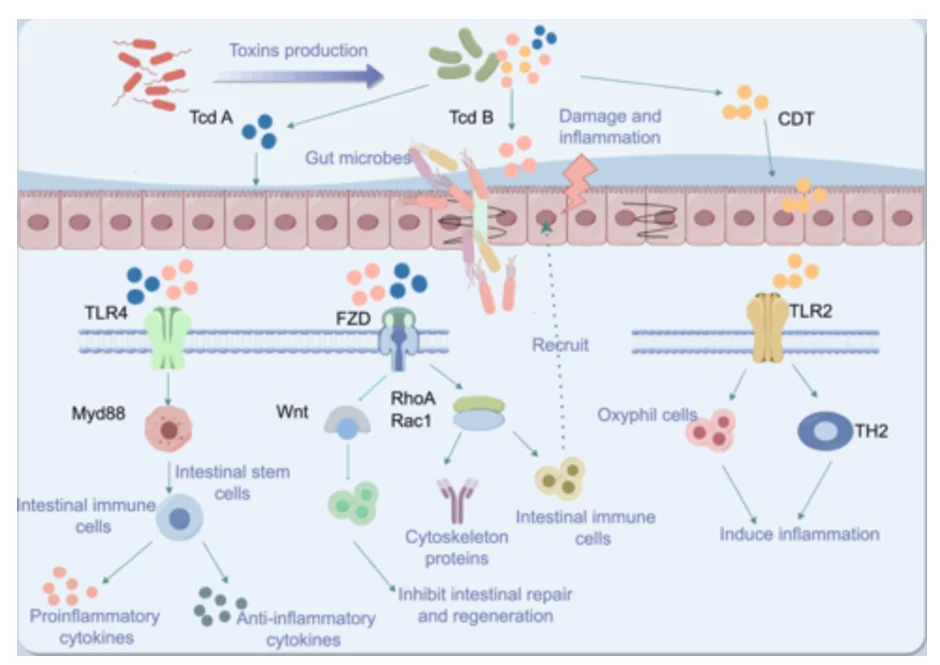
The pathogenesis of *Clostridium difficile* toxins and associated signaling pathways. *Clostridium difficile* produces three main toxins: Toxin A (TcdA), Toxin B (TcdB), and *Clostridium difficile* binary toxin (CDT). TcdA and TcdB are recognized by Toll‐like receptor 4 (TLR4), triggering an intestinal inflammatory response. Frizzled receptor (FZD) is a well‐established receptor for *Clostridium difficile* toxins, and its activation impedes intestinal damage repair. Additionally, CDT is a recently identified *Clostridium difficile* toxin, recognized by Toll‐like receptor 2 (TLR2), which induces intestinal inflammation. Myeloid differentiation primary response 88 (MyD88), Wnt family of proteins (Wnt), Ras homolog family member A (RhoA), Ras‐related C3 botulinum toxin substrate 1 (Rac1), T helper 2 (TH2).

Toll‐like receptor 2 (TLR2), an innate immune receptor located on the cell surface, mediates various immune responses, including protective type 2 immune responses orchestrated by T helper 2 (TH2) cells [76]. However, However, binary toxin (CDT) produced by certain *Clostridium difficile* exacerbates disease severity by activating TLR2. This activation indirectly induces eosinophil apoptosis and suppresses protective eosinophil and TH2 responses, further impairing immune defenses [77, 78]. Therefore, exploring and elucidating the common signaling pathways between *Clostridium difficile* toxins and inflammatory cells, as well as understanding how these toxins regulate this signaling network, will deepen our understanding of immune responses at both the cellular and molecular levels. This knowledge could contribute to the development of improved treatment strategies for CDI (Figure 2).

### Clostridium difficile and gut microbiota

#### Colonization resistance of the gut microbiome and CDI

The human gut harbors a complex microbiota comprising over 1000 species of bacteria, fungi, and protozoa that maintain a dynamic equilibrium in healthy individuals. The stability and composition of these microbial communities are critical for host health. Disruption of this balance, or dysbiosis, is a primary factor in the development of CDI [79]. This imbalance creates an ecological niche that promotes *Clostridium difficile* colonization [61].

Colonization resistance refers to the ability of the gut microbiota to prevent pathogenic organisms like *Clostridium difficile* from establishing and proliferating within the gastrointestinal tract. This phenomenon is a fundamental function of a healthy and diverse gut microbiota, achieved through a combination of physical, nutritional, and biochemical mechanisms. Healthy gut microbiota occupy niches along the intestinal lining, preventing pathogens from attaching to the mucosa. Current evidence suggests that the gut microbiota confer resistance through mechanisms such as spatial and nutrient competition and by regulating secondary metabolites [80]. However, the precise mechanisms through which the gut microbiota confer resistance to *Clostridium difficile* colonization remain incompletely understood. The prevailing perspective suggests that the gut microbiota primarily inhibit *Clostridium difficile* growth through spatial and nutrient competition, as well as by regulating secondary metabolites [2, 81].

#### Spatial and nutrient competition

Spatial competition arises when intestinal commensals inhibit pathogenic microbial adhesion to the mucosa. For example, *Lactobacillus rhamnosus* reduces *Clostridium difficile* adhesion by secreting extracellular polysaccharides, aiding in its clearance [82]. Nutrient competition further limits *Clostridium difficile* colonization as billions of gut microorganisms vie for limited resources. These interactions form the basis of colonization resistance, which involves complex microbial interactions that single‐strain interventions cannot replicate effectively [83].

A recent study reported that a non‐toxigenic *Clostridium difficile* strain effectively prevents rCDI by competitively inhibiting the germination of toxigenic *Clostridium difficile* through glycine consumption [8]. Similarly, *Saccharomyces boulardii* has been shown to reduce the adherence and colonization of *Clostridium difficile*, alleviate intestinal inflammation, and significantly decrease intestinal permeability [84]. Moreover, a clinical study indicated that *Saccharomyces boulardii* reduced the risk of CDI in elderly individuals [85]. Additionally, *Bifidobacterium longum* has been shown to reduce *Clostridium difficile* abundance and toxins, mitigating inflammation and enhancing survival in mice [86].

#### Gut microbiome changes in CDI patients

Studies have consistently shown that CDI is associated with reduced gut microbial diversity. In 2008, Chang et al. reported significantly lower fecal microbial diversity in CDI patients compared to healthy individuals (*p* = 0.0154) [87]. This finding was corroborated by a 2013 study, which also identified a reduced relative abundance of beneficial commensals from the *Ruminococcaceae* and *Lachnospiraceae* families and a decline in butyrate‐producing anaerobic fermenters [88]. Additionally, the abundance of *Bifidobacteria* and *Anaerobic Bacilli* genera negatively correlated with *Clostridium difficile* abundance, while increased *Enterococcus* abundance was linked to lower diversity and poorer clinical outcomes [89].

Recent findings have highlighted the role of gut fungi in CDI. Patients with CDI exhibited significantly lower alpha and beta diversity of gut fungi compared to healthy carriers, along with an increased abundance of *Cladosporium* and *Aspergillus* genera (*p* < 0.05). The ratio of *Ascomycota* to *Basidiomycota* was also significantly higher in CDI, suggesting fungal dysbiosis is an additional contributor to the disease [90].

### Clostridium difficile and gut metabolites

#### Clostridium difficile and bile acids

Bile acids, derived from gut microbiota metabolism, play a critical role in modulating *Clostridium difficile* colonization and infection. They are classified as primary or secondary based on their origin. Primary bile acids, including cholic acid (CA) and chenodeoxycholic acid (CDCA), are synthesized in the liver from cholesterol and conjugated with glycine or taurine. These are secreted into the small intestine, where intestinal microorganisms in the distal small intestine and colon convert them into secondary bile acids, such as deoxycholic acid (DCA) and lithocholic acid (LCA) [91].

**FIGURE 3:**
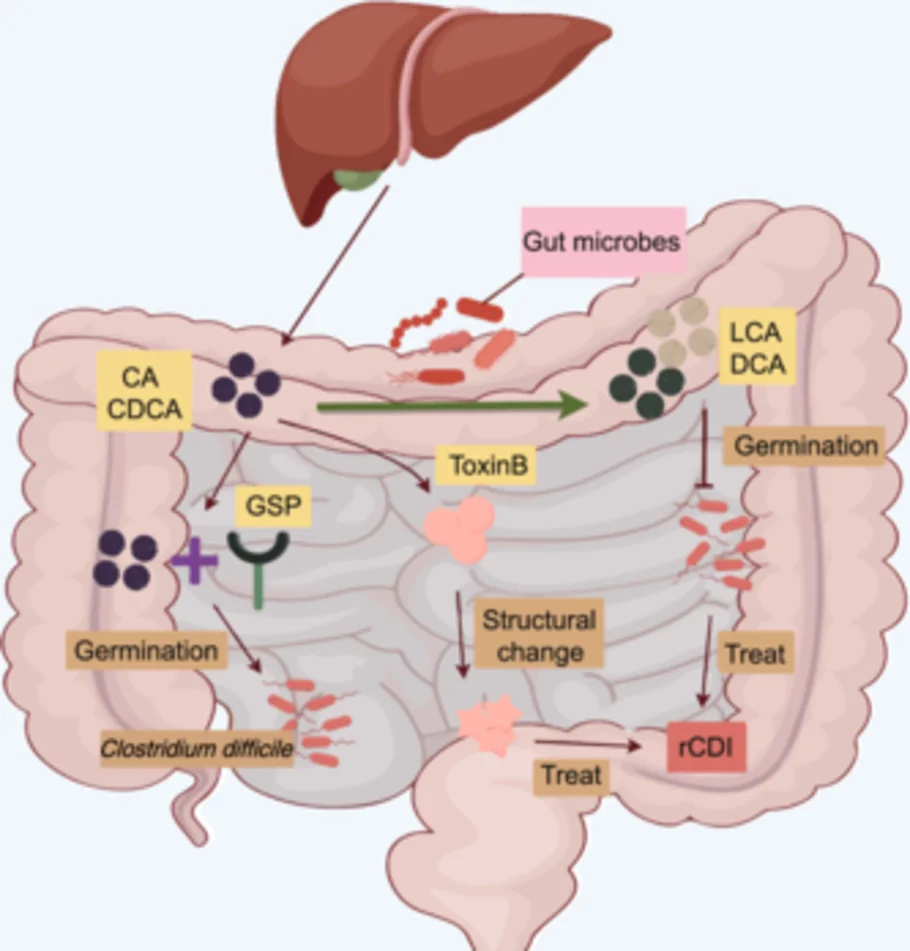
The liver secretes primary bile acids directly into the gastrointestinal tract, which can promote *Clostridium difficile* proliferation. Gut microbes convert primary bile acids into secondary bile acids, which inhibit *Clostridium difficile* growth and proliferation. Both primary and secondary bile acids can bind to Toxin B (TcdB), inducing structural changes and reducing its toxicity. CA, cholic acid; CDCA chenodeoxycholic acid; DCA, deoxycholic acid; GSP, graptolite signaling peptide; LCA, lithocholic acid; rCDI, recurrent *Clostridioides difficile* infection.

*Clostridium difficile* exploits bile acids as environmental cues for spore germination. While primary bile acids are generally nontoxic to *Clostridium difficile*, taurocholic acid specifically promotes spore germination through the receptor CspC [92, 93]. However, as the gut microbiota undergoes metabolic changes, secondary bile acids are produced, which exhibit toxicity towards *Clostridium difficile*. For example, ursodeoxycholic acid (UDCA) can inhibit spore germination in vitro and reduce recurrence rates [94]. Moreover, bacteria like *Clostridium scindens*, which possess bile acid 7‐dehydroxylation activity, enhance gut resistance to *Clostridium difficile* by increasing secondary bile acid production [95]. Notably, recent research has demonstrated that both primary and secondary bile acids can bind to TcdB, inducing structural changes that prevent TcdB from binding to cell surface receptors, thereby reducing its toxicity [96]. These findings are elaborated upon in Figure 3 and Table 2.

**TABLE 2:** The effects of bile acids on CDI in vitro and in vivo models.

Bile acids	Model	Treatment	The effects of bile acids in CDI	Reference
Desoxycholate	In vitro; mice	Supplement desoxycholate (1.9 × 10^−3^ M or 0.1%) to *Clostridium difficile* medium; cefoperazone induces CDI in mice.	Growth of *Clostridium difficile in vitro* ↑; germination of *Clostridium difficile in vitro* ↑; deoxycholate levels in CDI mice ↓.	[97]
Chenodeoxycholic acid	In vitro; mice	Supplement chenodeoxycholic acid (100 mM or 0.04%) to *Clostridium difficile* medium; mixed antibiotics induce CDI in mice.	Growth of *Clostridium difficile in vitro* ↓; germination of *Clostridium difficile in vitro* ↓; chenodeoxycholic levels in CDI mice ↓.	[98, 99, 100]
Lithocholic	In vitro; mice	Supplement lithocholic (0.1%) to *Clostridium difficile* medium; mixed antibiotics induce CDI in mice.	Growth of *Clostridium difficile in vitro* ↓; germination of *Clostridium difficile in vitro* ↓; lithocholic levels in CDI mice ↓.	[101, 102]
Taurocholate	In vitro; mice	Supplement taurocholate (0.1%) to *Clostridium difficile* medium; mixed antibiotics induce CDI in mice.	Germination of *Clostridium difficile in vitro* ↑; Germination of *Clostridium difficile in vivo* ↑; taurocholate levels in CDI mice ↑.	[103]
Glycocholate	In vitro	Supplement glycocholate (0.1%) to *Clostridium difficile* medium.	Germination of *Clostridium difficile in vitro* ↑.	[102]
Ursodeoxycholate	In vitro; mice	Supplement ursodeoxycholic acid (3.82 mM or 0.1%) to *Clostridium difficile* medium. Mixed antibiotics induce CDI in mice.	Growth of *Clostridium difficile in vitro* ↓; colonic bile acid structure ↑; colonic inflammatory responses in the early pathological process ↓.	[102, 104]

*Note*: “↑“ in the table means the quantity is increased or the effect is enhanced, “↓“ is the opposite. *Clostridioides difficile* infection (CDI).

Recent studies emphasize the potential of modifying bile acid composition as a therapeutic strategy for CDI. Secondary bile acids therapy CDI and recurrent CDI (rCDI) by inhibiting spore germination and attenuating toxin toxicity. Current research focuses on elucidating the underlying mechanisms and developing clinical treatments and nutritional strategies. These include improving bile acid composition through dietary or probiotic modulation of gut microbiota or developing specific bile acids and bile acid complexes as targeted therapeutic agents for CDI.

#### *Clostridium difficile* and short‐chain fatty acids (SCFAs)

**FIGURE 4:**
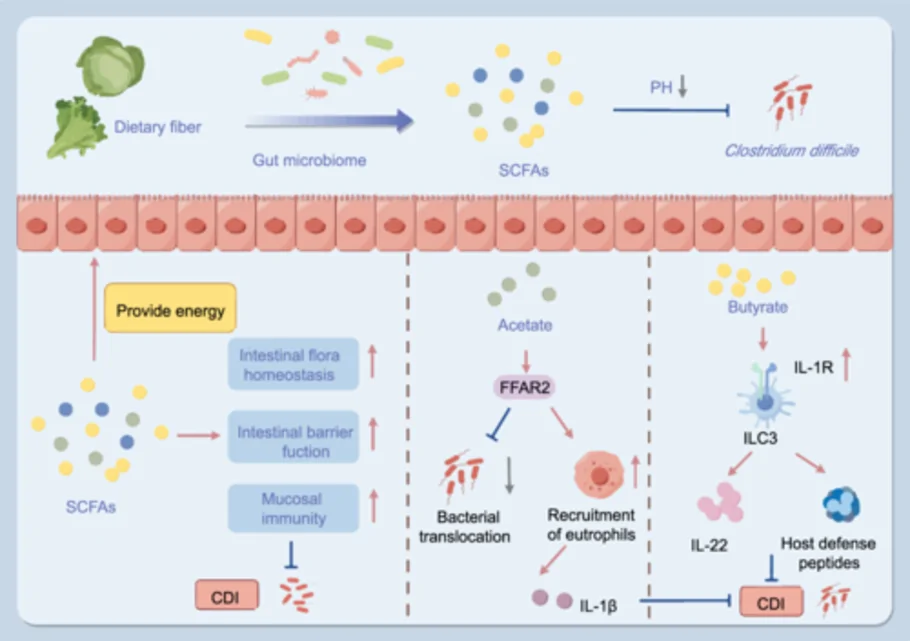
Gut microbes degrade dietary fiber into short‐chain fatty acids (SCFAs), which inhibit *Clostridium difficile* growth and reduce toxin production by lowering the gut pH. Furthermore, SCFAs regulate the host immune response, alleviating *Clostridium difficile* infection (CDI)by nourishing the gut, enhancing the intestinal microbiota, and strengthening the intestinal barrier. Acetate prevents *Clostridium difficile* translocation to extraintestinal organs by modulating Free fatty acid receptor 2 (FFAR2) expression. Additionally, acetate alleviates CDI by modulating FFAR2, promoting neutrophil recruitment to the site of inflammation, and inducing interleukin‐1 beta (IL‐1β) secretion. Conversely, butyrate alleviates CDI by enhancing interleukin‐1 receptor sensitivity on innate lymphoid cell type 3 (ILC3) cells, which induces interleukin‐22 (IL‐22) secretion and host defense peptide production.

Short‐chain fatty acids (SCFAs) are aliphatic monocarboxylic acids containing 2 to 8 carbon atoms produced in the colonic lumen by the microbial metabolism of carbohydrates and dietary fiber [105]. SCFAs, including acetate, butyrate, and valerate, play a critical role in gut health by supporting intestinal barrier function, mucosal immunity, and microbial homeostasis [106]. SCFAs serve as a significant energy source for colonocytes, maintaining colonic epithelial integrity and optimal nutrient absorption in the gut. Their anti‐inflammatory properties arise from modulating immune responses, reducing the production of pro‐inflammatory cytokines, and promoting the release of anti‐inflammatory mediators [106]. This anti‐inflammatory effect helps protect against gastrointestinal disorders, including IBD and CDI. In CDI, SCFAs appear to mitigate disease severity by supporting host immunity and maintaining gut barrier function. Patients with CDI exhibit significantly lower fecal SCFA levels than healthy individuals [107]. Similarly, a mouse model of CDI showed significantly reduced concentrations of SCFAs in the intestine and feces relative to healthy control mice [108]. SCFAs exert multiple protective mechanisms against CDI. They enhance immune function, promote the secretion of anti‐inflammatory cytokines [109], reduce toxin‐induced damage [110], and enhance tight junction integrity [97]. These findings are summarized in Figure 4 and Table 3. Notably, microbiota‐derived acetate activates the free fatty acid receptor 2 (FFAR2) on immune cells, enhancing neutrophil recruitment, stimulating IL‐1β secretion, and upregulating IL‐22 production by type 3 innate lymphoid cells (ILC3s) to combat CDI [98]. Butyrate exhibits similar effects, supporting neutrophil recruitment to the colon's lamina propria and stimulating the expression of HDPs (e.g., regenerating islet‐derived protein 3β (Reg3β) and Reg3γ) and cytokines that contribute to immune responses against CDI [99]. Valerate, another SCFA, has shown specific efficacy against CDI. FMT treatment in CDI patients significantly increased valerate levels, correlating with symptom improvement. *In vitro* studies revealed that valerate selectively inhibits *Clostridium difficile* growth without affecting beneficial gut bacteria such as *Bacteroidetes spp*, *Clostridium* scindens, and *Firmicutes spp* [100].

**TABLE 3:** The effects of SCFAs on CDI in vitro and in vivo models.

SCFAs	Model	Treatment	The effects of SCFAs on CDI	Reference
Potassium acetate	In vitro; mice	TcdA (30 nM/mL) injected into the ileal loops; Potassium acetate (100 µM/ml) injected into the ileal loops.	Production of IL‐6 ↓ ; HDAC‐6 activity ↓; α‐tubulin acetylation ↑; inflammatory response and villus disruption ↓.	[111]
Acetate	Mice	The addition of 150 mM acetate to the drinking water of mice; 5% fiber supplement in the diet; CDI.	Histopathological scores ↓; body weight ↑; the levels of IL‐1B and IL‐22 ↑; the level of SCFAs in the intestinal tract ↑.	[76]
Valerate	Human; mice	Recurrent CDI patients; CDI mice; gavaged with 200 µL of 15 mM glycerol trivalerate; clindamycin‐induced rCDI mice; FMT.	Histopathological scores ↓; body weight ↑; valerate concentrations ↑; *C difficile* TVC ↓; total bacterial biomass ↓.	[112]
Butyrate	Mice	CDI; The addition of 150 mM butyrate to the drinking water of mice.	Fecal bacterial load ↓; microbial diversity ↓; expression of pro‐inflammatory cytokines ↓; expression of HIF‐1 ↑.	[113]
Butyric acid	In vitro	A mixture of butanol or butyric acid extracted in vitro and *Clostridium difficile*.	Toxins production of *Clostridium difficile* in medium expression of toxins production in SDM ↑.	[114]
Volatile Fatty Acids	*In vitro*; hamster model	A mixture of fatty acids extracted in vitro and *Clostridium difficile*.	The in vitro growth of *Clostridium difficile* ↓; *Clostridium difficile* activity ↓.	[115]

*Note*: “↑“ in the table means the quantity is increased or the effect is enhanced, “↓“ is the opposite. Short‐chain fatty acids (SCFAs); *Clostridioides difficile* infection (CDI); interleukin‐6 (IL‐6); histone deacetylase 6 (HDAC‐6); interleukin‐1B (IL‐1B); interleukin‐6 (IL‐22); total viable count (TVC); fecal microbiota transplantation (FMT); hypoxia‐inducible factor 1 (HIF‐1); site‐directed mutagenesis (SDM).

Additionally, SCFAs contribute to gut acidification, a critical factor in CDI management. CDI patients often exhibit an alkaline gut environment, which fosters *Clostridium difficile* growth and toxin activity. Fermentable fiber, by stimulating SCFA production, lowers gut pH and creates an unfavorable environment for *Clostridium difficile*, while enhancing toxin degradation [101, 102].

#### Clostridium difficile and amino acids

Amino acids, derived from enzymatic digestion of dietary proteins, are essential nutrients for gut health, playing critical roles in intestinal growth, mucosal integrity, and barrier function. [103]. However, they also significantly influence the germination, growth, and metabolism of *Clostridium difficile*, a pathogen implicated in severe gastrointestinal infections. Essential amino acids, including cysteine, isoleucine, leucine, proline, tryptophan, and valine, are necessary for *C. difficile* growth. Non‐essential amino acids such as arginine, glycine, histidine, methionine, and threonine, while not indispensable, can enhance bacterial proliferation. [104]. Amino acids also facilitate *Clostridium difficile* spore germination; for example, taurocholic acid and glycine are key stimulatory factors. Histidine, in conjunction with glycine and taurocholate, has been identified as a co‐factor in promoting spore germination [26, 111].

Amino acids also modulate *Clostridium difficile* pathogenicity. For example, cysteine inhibits the mRNA expression of *Clostridium difficile* toxins TcdA and TcdB. Additionally, several energy‐related metabolic pathways, such as butyrate metabolism, the Wood‐Ljungdahl pathway, Stickland reactions, and the ATP synthase complex, are suppressed in the presence of cysteine [116]. Conversely, high concentrations of isoleucine (100 mM) enhance TcdA and TcdB production [113]. Proline, an essential energy source for *Clostridium difficile*, downregulates toxin production by inhibiting TcdR when present in excess [117]. Arginine supports *Clostridium difficile* growth, but its absence may impair bacterial proliferation while increasing toxin production in certain strains [118]. Tryptophan and its derivatives also interact with host metabolism, potentially mitigating *Clostridium difficile* immunopathology by modulating host tryptophan catabolism [111]. The interplay between amino acids and CDI is summarized in Table 4.

**TABLE 4:** The effects of amino acids on CDI in vitro and in vivo models.

Amino acids	Model	Treatment	The effects of amino acids on *Clostridium difficile*	Reference
Cysteine	In vitro	Supplement cysteine (0.5 g/L) to *Clostridium difficile* medium.	The production of toxins ↑.	[119, 120]
Isoleucine	In vitro	Supplement isoleucine (100 mM) to *Clostridium difficile* medium.	The production of toxins ↑.	[121]
Arginine	In vitro	Supplement arginine (0.1 g/L) to *Clostridium difficile* medium; complete exclusion of arginine from the medium	Growth of *Clostridium difficile* ↑; The production of toxins ↓.	[122]
Leucine	In vitro	Supplement leucine (0.2 g/L) to *Clostridium difficile* medium; complete exclusion of leucine from the medium	The production of toxins ↓; Growth of *Clostridium difficile* ↑.	[121, 122]
Proline	In vitro	Supplement proline (0.3 g/L) to *Clostridium difficile* medium.	The production of toxins ↓.	[121]
Threonine	In vitro	Supplement threonine (0.2 g/L) to *Clostridium difficile* medium.	Growth of *Clostridium difficile* ↑.	[26]
Valine	In vitro	Complete exclusion of leucine from the medium; supplement valine (0.3 g/L) to *Clostridium difficile* medium.	Growth of *Clostridium difficile* ↑; The production of toxins ↓.	[121]
Glycine	In vitro	Supplement glycine (0.1 g/L) to *Clostridium difficile* medium.	Growth of *Clostridium difficile* ↑.	[26]
Methionine	In vitro	Supplement methionine (0.2 g/L) to *Clostridium difficile* medium.	Growth of *Clostridium difficile* ↑.	[121]
Tryptophan	IDO1−/− C57BL/6 mice	Cefoperazone induce CDI in mice.	The number of neutrophils ↓. The inflammatory response in mice ↓.	[123]

*Note*: “↑“ in the table means the quantity is increased or the effect is enhanced, “↓“ is the opposite. *Clostridioides difficile* infection (CDI).

While amino acids contribute to *Clostridium difficile* growth and complicate CDI treatment, they also hold potential therapeutic value due to their enteric nutritional properties. For example, tryptophan and its derivatives may regulate host immune responses to infection. These dual roles underscore the need for further research to elucidate the in vivo effects of amino acid metabolism on *Clostridium difficile* and to develop targeted nutritional strategies for managing CDI.

## 4 DIET AND GUT HEALTH IN CDI

### Dietary meat, protein, and gut microbiota in CDI

The Western diet (WD), prevalent in Northern Europe and the USA, is characterized by high intake of red and processed meats, high‐fat dairy products, high‐sugar beverages, refined grains, and butter, and is often referred to as the “meat‐sweet” diet [124]. Studies indicate that *Clostridium difficile* is frequently detected in livestock and poultry products, suggesting that red meat consumed in human diets may act as a reservoir for this pathogen [112, 114]. Additionally, red meat in the WD serves as a major source of bioavailable zinc. Excessive dietary zinc has been shown to disturb gut microbes, reducing the antibiotic dose required to increase susceptibility to CDI. Zinc also exacerbates *Clostridium difficile* severity by enhancing toxicity, reducing Rho activity in the intestinal epithelium, and triggering a severe immune response [115]. A high‐fat, high‐protein diet further worsens CDI symptoms and increases mortality in mice [21] by providing abundant energy sources for *Clostridium difficile* through the Stickland reaction, where amino acids and peptides are metabolized. Proline and glycine, key Stickland acceptors [115], are generated in greater quantities in the intestinal lumen due to high dietary protein and fat, favoring *Clostridium difficile* overgrowth [21].

The WD's low fiber and high‐fat content alters bile acid metabolism. High‐fat intake increases primary bile acids while reducing secondary bile acids produced by the gut microbiota, promoting *Clostridium difficile* proliferation. Additionally, the WD reduces gut microbial abundance and diversity, decreasing colonization resistance to *Clostridium difficile* [125]. The lack of dietary fiber and polyunsaturated fatty acids (PUFAs) in WD lowers SCFA levels, which play a protective role in gut health [126, 127]. Therefore, WD increases the incidence of CDI by decreasing gut microbiota abundance and colonization resistance, while also reducing SCFA levels, resulting in more severe intestinal damage.

### Dietary fiber and gut microbiota in CDI

Ample scientific evidence supports the beneficial effects of fiber on intestinal health [128]. Fiber supplementation, particularly with pectin, enhances microbial diversity, mitigates colonic inflammation, and promotes the production of SCFAs and chenodeoxycholic acid (CDCA), both of which help resist *Clostridium difficile* colonization [129]. A previous study demonstrated that dietary inulin increased fecal microbiota load and density while reducing the abundance of *Clostridium difficile* in a mouse model [121]. Additionally, dietary xanthan gum alleviated CDI‐induced diarrhea and intestinal inflammation by preserving microbiota diversity and abundance during antibiotic treatment, resisting *Clostridium difficile* colonization, and increasing SCFA concentrations [14].

### Dietary polyphenols and gut microbiota in CDI

Plant‐derived polyphenols bind to bacterial cell membranes in a dose‐dependent manner, disrupting bacterial functions, inhibiting cell growth, reducing spore production, and modulating related genes [130]. For example, tea polyphenols are metabolized by bacteria to produce metabolites that effectively inhibit *Clostridium difficile* growth in fecal cultures [131]. Several studies have shown the effects of specific polyphenols on *Clostridium difficile*. Such as at subinhibitory concentrations (0.5 mg/mL), carvacrol significantly reduces spore formation and downregulates genes essential for spore production. When combined with trans‐cinnamaldehyde, carvacrol further suppresses toxin production (TcdA and TcdB), thereby reducing cytotoxicity [50, 132]. In fecal suspensions of pure *Clostridium difficile* cultures, thymol effectively inhibits bacterial growth at concentrations of 0.1 and 0.5 mg/mL, with higher concentrations showing greater efficacy [122]. Epigallocatechin‐3‐gallate (EGCG), a key bioactive component of green tea, EGCG modulates the gut microbiota by reducing the relative abundance of *Enterococcaceae*. It also improves amino acid metabolism, alleviates colonic inflammation, increases body weight, and reduces mortality in a CDI mouse model [123]. Supplementation with carvacrol improves clinical outcomes in CDI mice by shifting gut microbiota composition. It increases beneficial bacteria such as *Phyllostachys spp*. and decreases harmful bacteria such as *Aspergillus spp*. and *Clostridium difficile* [119].

Dietary polyphenols show significant potential for preventing and treating CDI. However, further research is needed to fully understand the mechanisms behind the synergy between dietary fiber and polyphenols in promoting gut health. Clinical validation will be crucial for translating these findings into practical dietary strategies for preventing and treating CDI.

### Specific diet therapy in CDI

Dietary interventions, such as the Crohn's Disease Exclusion Diet (CDED) and the Specific Carbohydrate Diet (SCD), are gaining attention as potential strategies for managing CDI. These approaches aim to modulate the gut microbiota, strengthen intestinal barrier function, and minimize immune system dysregulation by avoiding harmful dietary components. The CDED is a whole‐food‐based approach that eliminates processed foods and food additives, aiming to avoid adverse effects on the immune system, gut microbiota, and intestinal barrier function. Food additives, widely used in the food industry to enhance taste, texture, or appeal, can negatively impact the composition and diversity of the gut microbiota, even at regulatory‐approved doses [120]. For instance, trehalose, a food additive long deemed safe, has been shown to increase toxin expression in certain highly virulent *Clostridium difficile* strains (RT027 and RT078). This has been associated with more severe disease manifestations and higher mortality rates in CDI mouse models. Research by Collins et al. revealed that these strains have evolved efficient mechanisms to metabolize trehalose, potentially driving their prevalence in recent epidemic outbreaks [133]. Consequently, the widespread adoption of trehalose in food production has been linked to the dominance of these virulent strains in the 21st century. Notably, while trehalose metabolism provides a growth advantage to *Clostridium difficile* strains in specific conditions, studies using an in vitro intestinal model found that supplementation with trehalose, glucose, or saline did not significantly alter toxin production or spore formation [134]. These findings suggest that the relationship between trehalose and *Clostridium difficile* virulence may depend on complex environmental or host factors.

Although the effects of food additives on CDI remain understudied, adopting a CDED, which excludes additives, may be a prudent precautionary measure until more definitive evidence emerges. The SCD, which restricts the intake of complex carbohydrates such as disaccharides and polysaccharides while permitting only monosaccharides, offers another dietary approach. In addition to carbohydrate restriction, the SCD includes high‐quality proteins, fiber‐rich fruits and vegetables, and healthy fats while excluding processed carbohydrates such as wheat, barley, corn, and rice [135].

Disaccharides in the gut are fermented by bacteria to produce organic acids such as lactic acid, carbon dioxide, and nitrogen gas. When disaccharides remain unabsorbed, they increase osmotic pressure in the intestinal lumen, impairing water absorption and leading to diarrhea. The organic acids can irritate the intestinal tract, causing acidic stools and distention due to excessive gas production. Unlike harmful bacteria that utilize disaccharides for metabolism, SCD aims to limit bacterial and fungal overgrowth by promoting monosaccharide metabolism, which supports healthy gut microbiota while limiting bacterial and fungal overgrowth [136].

In clinical trials, Suskind et al. reported that three pediatric patients with CDI or recurrent CDI (rCDI) showed increased intestinal microbial diversity and a reduction in *Clostridium difficile* abundance after following the SCD. Notably, one patient with rCDI was completely cured through dietary intervention [137]. Despite these promising findings, it is crucial to gather robust scientific evidence on the effects of CDED and SCD on CDI and their underlying mechanisms before recommending these diets as standard treatments.

## 5 CONCLUSION AND FUTURE TRENDS

CDI remains a significant healthcare challenge worldwide, demanding innovative solutions for effective management. This review has explored the pathological mechanisms underlying CDI and the role of dietary patterns in influencing its progression. Addressing CDI requires a multifaceted approach, including interrupting the infection cycle, mitigating the effects of *Clostridium difficile* toxins, and restoring a balanced gut microbiota. Despite advancements in understanding these processes, further research is needed to clarify the underlying mechanisms and develop targeted therapeutic strategies. One promising approach lies in leveraging colonization resistance, which fosters a resilient and diverse gut microbiota. By promoting beneficial bacterial populations, this approach creates an environment less conducive to *Clostridium difficile* colonization and growth, thereby reducing infection risk. Dietary interventions are critical in this context, yet a universally accepted dietary regimen for managing CDI is still lacking.

Future advancements in precision nutrition, nutrigenomics, systems biology, and bioinformatics, hold great promise for creating personalized dietary interventions. These interventions can be tailored to account for the variations in gut microbiota composition across diverse patient populations with CDI. Furthermore, a comprehensive database has been established to organize macro‐genomic and metabolomic data obtained from stool samples of CDI patients. This database can facilitate the prediction of patient outcomes and guide the development of appropriate dietary strategies at various stages of infection. By combining innovative research with personalized approaches, the field is poised to ultimately advance the prevention and management of CDI.

## AUTHOR CONTRIBUTIONS

**Hao Yang**: Conceptualization; writing—original draft; writing—review and editing. **Lamei Wang**: Conceptualization; writing—review and editing. **Javier A Villafuerte Gálvez**: Writing—review and editing. **Xinru Ma**: Visualization. **Hua Xu**: Writing—review and editing. **Shengru Wu**: Writing—review and editing. **Junhu Yao**: Writing—review and editing. **Xinhua Chen**: Writing—review and editing. **Yangchun Cao**: Conceptualization; writing—review and editing; project administration.

## CONFLICT OF INTEREST STATEMENT

The authors declare no conflicts of interest.

## 1 ETHICS STATEMENT

No animals or humans were involved in this study.

## Data Availability

Data sharing is not applicable to this article as no datasets were generated or analyzed during the current study. No new data and scripts were generated for this review. Supplementary information (graphical abstract, slides, videos, Chinese translated version, and update materials) may be found in the online DOI or iMeta Science http://www.imeta.science/imetaomics/.
